# The MONARCH intervention to enhance the quality of antenatal and postnatal primary health services in rural South Africa: protocol for a stepped-wedge cluster-randomised controlled trial

**DOI:** 10.1186/s12913-018-3404-3

**Published:** 2018-08-08

**Authors:** Terusha Chetty, H. Manisha N. Yapa, Carina Herbst, Pascal Geldsetzer, Kevindra K. Naidu, Jan-Walter De Neve, Kobus Herbst, Philippa Matthews, Deenan Pillay, Sally Wyke, Till Bärnighausen, for the MONARCH study team

**Affiliations:** 1 Africa Health Research Institute, Somkhele, P.O. Box 198, Mtubatuba, KwaZulu-Natal 3935 South Africa; 2 Department of Global Health and Population, Harvard T. H. Chan School of Public Health, 677 Huntington Avenue, Boston, MA 02115 USA; 3 Maternal Adolescent and Child Health Systems (MatCH), School of Public Health, University of Witswatersrand, Braamfontein, South Africa; 4 Institute of Public Health, Heidelberg University, Im Neuenheimer Feld 324, 69120 Heidelberg, Germany; 5 Division of Infection & Immunity, University College London, Gower Street, Bloomsbury, London, WC1E 6BT UK; 6 Institute for Health and Wellbeing, University of Glasgow, 1 Lilybank Gardens, Glasgow, G12 8RZ Scotland, UK; 7 Department of Global Health, University College London, Gower Street, Bloomsbury, London, WC1E 6BT UK

**Keywords:** Health systems, Continuous quality improvement, Maternal, HIV, Stepped wedge, Randomised trial

## Abstract

**Background:**

Gaps in maternal and child health services can slow progress towards achieving the Sustainable Development Goals. The Management and Optimization of Nutrition, Antenatal, Reproductive, Child Health & HIV Care (MONARCH) study will evaluate a Continuous Quality Improvement (CQI) intervention targeted at improving antenatal and postnatal health service outcomes in rural South Africa where HIV prevalence among pregnant women is extremely high. Specifically, it will establish the effectiveness of CQI on viral load (VL) testing in pregnant women who are HIV-positive and repeat HIV testing in pregnant women who are HIV-negative.

**Methods:**

This is a stepped-wedge cluster-randomised controlled trial (RCT) of 7 nurse-led primary healthcare clinics to establish the effect of CQI on selected routine antenatal and postnatal services. Each clinic was a cluster, with the exception of the two smallest clinics, which jointly formed one cluster. The intervention was applied at the cluster level, where staff received training on CQI methodology and additional mentoring as required. In the control exposure state, the clusters received the South African Department of Health standard of care. After a baseline data collection period of 2 months, the first cluster crossed over from control to intervention exposure state; subsequently, one additional cluster crossed over every 2 months. The six clusters were divided into 3 groups by patient volume (low, medium and high). We randomised the six clusters to the sequences of crossing over, such that both the first three and the last three sequences included one cluster with low, one with medium, and one with high patient volume.

The primary outcome measures were (i) viral load testing among pregnant women who were HIV-positive, and (ii) repeat HIV testing among pregnant women who were HIV-negative. Consenting women ≥18 years attending antenatal and postnatal care during the data collection period completed outcome measures at delivery, and postpartum at three to 6 days, and 6 weeks. Data collection started on 15 July 2015. The total study duration, including pre- and post-exposure phases, was 19 months. Data will be analyzed by intention-to-treat based on first booked clinic of study participants.

**Discussion:**

The results of the MONARCH trial will establish the effectiveness of CQI in improving antenatal and postnatal clinic processes in primary care in sub-Saharan Africa. More generally, the results will contribute to our knowledge on quality improvement interventions in resource-poor settings.

**Trial registration:**

This trial was registered on 10 December 2015: www.clinicaltrials.gov, identifier NCT02626351.

**Electronic supplementary material:**

The online version of this article (10.1186/s12913-018-3404-3) contains supplementary material, which is available to authorized users.

## Background

South Africa has made considerable gains in improving prevention of mother-to-child transmission of HIV (PMTCT) services coverage since being identified as a Global Plan Priority country in 2009 [1]. In 2012, nearly 4 million women in the country lived with HIV and antenatal HIV prevalence was 30% [2]. The PMTCT Cascade of Care (CoC) refers to key steps in the clinical pathway from early diagnosis of HIV in pregnancy/postpartum to achieving long-term maternal HIV virologic suppression on antiretroviral therapy (ART), retention-in-care, and early infant diagnosis and treatment [3]. Leakages in the PMTCT care cascade hinder progress towards eliminating paediatric HIV and protecting maternal and child health.

Among pregnant women who are HIV-positive, HIV viral load (VL) testing is critical to judge the clinical performance of ART and to prevent vertical transmission of HIV from mother to child [4, 5]. Vertical transmission risk increases with maternal VL [6, 7]. VL testing can identify virologic failure early, leading to timely interventions such as adherence counselling, resistance testing and treatment switches, and enhanced antiretroviral prophylaxis for HIV-exposed infants [8]. Moreover, VL testing is important because large proportions of pregnant women on ART are failing virologically (by some estimates 20–40% [9, 10]). The South African National PMTCT guidelines recommend universal ART for pregnant women who are HIV-positive and regular VL testing to monitor the clinical performance of ART [11]. However, in many communities in South Africa and other sub-Saharan African countries the majority of pregnant women on ART, and non-pregnant women and men, do not receive regular VL tests [12–15].

Repeat HIV testing of pregnant women who are HIV-negative is important because HIV incidence in pregnancy is high in many countries [16]. In South Africa, HIV incidence in pregnancy and postpartum is estimated to be above 4% [10, 16]. Since VL increases markedly shortly after infection, the risk of MTCT is particularly high among recently infected women [17]. Early diagnosis of HIV and initiation of ART are thus critical to ensure that MTCT is eliminated, but this requires pregnant women who are HIV-negative to be re-tested at regular intervals [18]. The South African National PMTCT guidelines thus recommend pregnant women who are HIV-negative to be tested in three-monthly intervals after the initial HIV test during the first antenatal visit [11]. However, in South Africa and other countries in sub-Saharan Africa large proportions of pregnant women who are HIV-negative do not receive repeat HIV tests [17, 19, 20].

In addition to the need for improvements in early diagnosis of HIV and regular VL monitoring of ART in antenatal care, adherence to clinical guidelines and quality of care continue to be major challenges following delivery in many countries [21, 22], undermining the delivery of effective postnatal care to mothers and children including ART, safe infant feeding and postnatal contraception.

One important approach to improving quality of care in the health sector is Continuous Quality Improvement (CQI) [23, 24]. Several features of CQI make it an attractive choice to improve quality of care and adherence to clinical guidelines in pregnancy-related care in sub-Saharan Africa. First, CQI uses time-tested and robust management techniques to diagnose quality problems, develop solutions and monitor progress. These techniques can be adapted to improve many different processes and healthcare functions, depending on clinical contextual needs and opportunities for improvement identified with real-time data [23, 24]. Second, CQI empowers health workers to develop approaches and take actions to improve quality of care on an ongoing basis. CQI mentors work with local clinic staff to facilitate the development of solutions to quality of care shortcomings that the staff identify as best suited to their local contexts. Third, CQI works within existing resource constraints and does not require large long-term investments to ensure sustained improvements in quality of care [25].

Several randomized controlled trials have demonstrated that CQI initiatives can be successful in improving quality of hospital care in resource-rich countries [26–28]. However, despite major CQI initiatives in primary care in several sub-Saharan African countries [29–33], rigorous scientific evidence on CQI effectiveness in these settings is largely lacking.

The MONARCH stepped-wedge cluster RCT will for the first time establish the effectiveness of an intervention to improve VL testing in pregnant women who are HIV-positive and repeat HIV testing in pregnant women who are HIV-negative in primary care clinics in rural South Africa.

More broadly, the trial will contribute to our understanding of the effectiveness of CQI in improving the quality of antenatal and postnatal care and add to the scarce but growing evidence on the effectiveness of CQI as an approach to improve quality of care in sub-Saharan Africa [34, 35]. This evidence is important because several governments in sub-Saharan Africa are using CQI to improve quality of care in public-sector healthcare provision [29–33].

### Primary and secondary endpoints

Our primary endpoints are (i) viral load testing among pregnant women who are HIV-positive and (ii) repeat HIV testing among pregnant women who are HIV-negative. Our secondary endpoints are:

(i) Patient experiences of healthcare quality among all pregnant women
(ii) Maternal health outcomes including:
  - Postnatal care attendance within 6 weeks postpartum among all pregnant women
  - Maternal retention in HIV care among women who are HIV-positive
  - ART utilization among women who are HIV-positive
  - Maternal HIV virologic suppression among women who are HIV-positive
  - HIV seroconversion in pregnancy among women who are HIV-negative
(iii) Infant health outcomes including:
  - Mother-to-child transmission of HIV
  - Nevirapine prophylaxis for HIV-exposed infants
  - Infants with weight, length, and head circumference measured
(iv) Maternal knowledge and uptake of key services including:
  - HIV services
  - Contraceptive services
  - Exclusive breastfeeding
(v) Healthcare provider job satisfaction and motivation

## Methods and design

### Study setting and CQI implementation

**Fig. 1:**
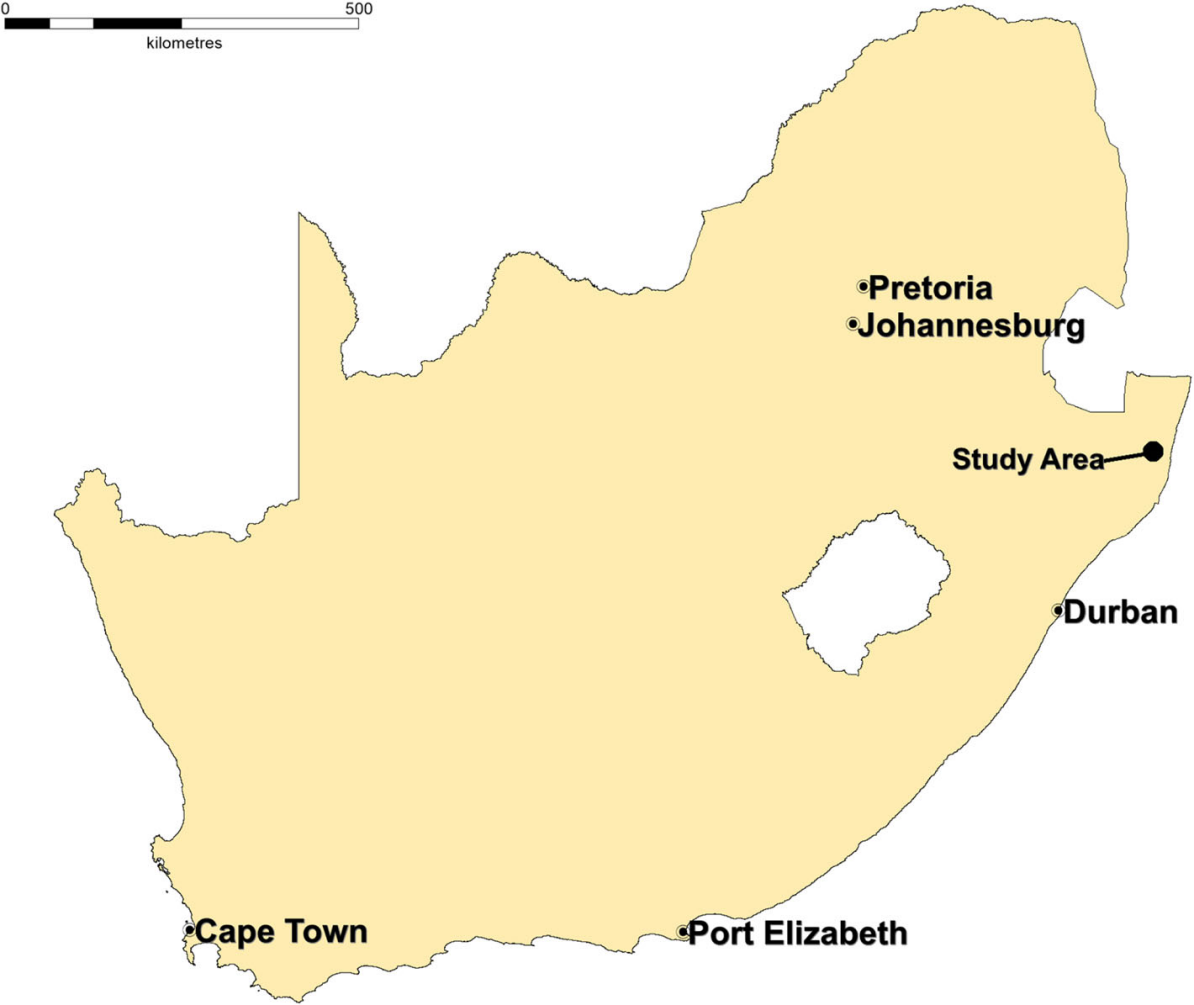
The Africa Health Research Institute study site at Somkhele. Location of the MONARCH study. Based on: Tanser et al. 2008 [46]

The study setting is rural KwaZulu-Natal, South Africa, in the community participating in the population health research carried out by the Africa Health Research Institute (AHRI) which was previously known as the Africa Centre for Health and Population Studies. AHRI is located within a 438 km^2^ area in the mostly rural Hlabisa sub-district of northern KwaZulu-Natal (Fig. 1). As a Wellcome Trust-Howard Hughes Medical Institute major overseas programme, the AHRI Population Intervention Platform Surveillance Area (PIPSA) South has collected comprehensive longitudinal population and HIV surveillance data on consenting individuals ≥15 years old (approximately 90,000 people in 11,000 households) since 2003. HIV prevalence amongst women of reproductive age is ~ 37% [36]. Overall fertility has been stabile since 2000, at about three children per woman, with an average of 2200 live births per year [37].

There are 6 nurse-led Department of Health (DoH) primary healthcare clinics (PHCs) of varying size within the geographic bounds of PIPSA South. The clinic immediately outside the PIPSA South geographic bounds located in the market town of Mtubatuba, adjoins a major highway and is often visited by PIPSA residents regardless of proximity. It was thus added to the 6 clinics within PIPSA South for this study. Oversight of these 7 clinics is led by the local primary level sub-district hospital, Hlabisa Hospital. Medical officer support is provided weekly where possible.

The CQI intervention in this study was delivered by the University of KwaZulu-Natal (UKZN) Centre for Rural Health (CRH) CQI specialist team who travelled from Durban, KwaZulu-Natal, to the study site. They worked collaboratively with PHC staff to implement changes in clinical practice through training and mentorship using real-time clinic data. The CRH team consisted of two isiZulu-speaking South African professional nurses (CRH CQI mentors) and a data capturer who carried out field activities, with close support from an Improvement Advisor (a consultant obstetrician with CQI experience), a Scientific Advisor and a data manager. CQI field activities for the entire project were scheduled in advance of the first intervention step and are described in detail below.

### Study design

**Fig. 2:**
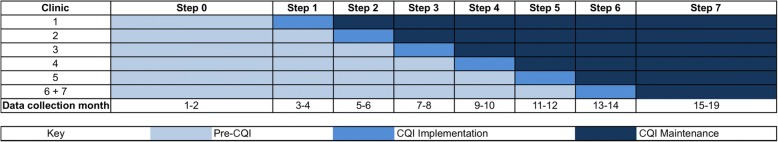
The MONARCH stepped wedge study design. Clinics provided baseline (pre-intervention) data until each rolled over to the intervention in random order. All clinics provided data continuously throughout the study period. Baseline data collection across all clinics occurred from 15 July 2015 to 28 September 2015 (Step 0). As data extraction on antenatal visits was retrospective from the point of delivery, the baseline observation period covered an additional ~ 6 months for the first recruited participants – thus Step 0 covered a duration of ~ 8 months

The study, a stepped-wedge cluster RCT, was carried out from 15 July 2015 to 30 January 2017, at the 7 DoH PHCs described above: Mtubatuba, KwaMsane, Mpukunyoni, Somkhele, Machibini, Esiyembeni and Gunjaneni. All clusters crossed over from control to intervention exposure according to the stepped-wedge study design (Fig. 2). The six clusters were randomised to the calendar sequences of crossing over. The stepped-wedge study design was selected because (i) it was considered unethical to withhold the intervention from some clinics as CQI has known efficacy in resource-rich settings; (ii) the participation of all clinics both during the control and the intervention exposure state was thought to be better if it was known that during the course of the trial all clinics would receive the intervention; (iii) the field implementers of CQI were a small team of three people, making simultaneous rollout in all clusters impracticable; and (iv) it allows adjustment for secular trends in outcomes. Data collection occurred at all 7 DoH PHCs and Hlabisa Hospital maternity ward as the majority of deliveries from the sub-district occur at Hlabisa Hospital.

### Eligibility criteria

#### Clusters

The clusters were pre-defined and included the 6 PHCs within the PIPSA South area plus Mtubatuba clinic. Antenatal care providers within clusters participated in the CQI activities based on availability. Efforts were made to recruit providers in leadership roles (e.g. Operational Managers, Professional Nurses) to increase likelihood of sustainability of the intervention and dissemination of skills. Based on clinic size (and staffing) approximately five participants from each clinic constituted a facility-level CQI team, which ideally included at least one person in a leadership position.

#### Individuals

All women aged 18 years or older were overall eligible for outcome and exposure assessment at three time points independent of previous or prospective recruitment status: at delivery, at the 3–6 day postnatal visit, and at the 6-week postnatal visit. Recruitment occurred continuously at all clinics and Hlabisa Hospital over the entire study period regardless of the clinic’s randomisation status. Women who had just delivered a baby at Hlabisa Hospital or any of the 7 study clinics were recruited for outcome and exposure assessment if they had attended any of the 7 study clinics for antenatal care (ANC) or resided in PIPSA South during their pregnancy. Consenting women attending a 3–6 day or 6-week postnatal visit at a study clinic with their infant (aged < 8 days or 5–8 weeks respectively) were recruited regardless of ANC clinic attended or where they resided during pregnancy.

### Procedures

#### Conceptual framework

We hypothesized that this CQI intervention would bring about the desired changes in clinical processes by providing a supportive and motivating environment alongside real-time data to PHC-level healthcare providers. The CRH team supported PHC staff to implement simple workable solutions to gaps identified through collaborative root-cause analyses. Furthermore, CQI may have increased job satisfaction and empowerment through reduced workload and better patient outcomes. Implicit in these assumptions were staff availability to participate in CQI activities and the ability to continue normal clinical duties in parallel to CQI activities.

#### Intervention package

Each intervention step was of 2 months’ duration with a 2-month pre-exposure data collection period and a 4.5-month endline (Fig. 2). We refer to the first 2-month intervention step as the ‘transition’ phase – this was the start of the intervention, in which ‘intensive’ CQI was delivered. The maintenance phase occurred after this transition phase for each cluster and included support and maintenance CQI visits in approximately monthly intervals. Visit types and planned activities are described in Table 1.

**Table 1:** Description of CQI intervention visit types and activities

Study phase	Visit type	Description of activities
Intensive CQI (transition)	Induction	• Introduction to the CRH QI team • Situational analysis with preliminary appraisal of local clinic processes and pre-exposure data collection using facility data sources such as registers • Training of facility staff by CRH CQI mentors on CQI methodology and tools
	Intervention	• Mapping clinic processes and establishing priorities for process improvements through bottleneck and root-cause analyses with standardized CQI tools such as process maps, fishbone diagrams, initiation of PDSA cycles and run charts • Implementing changes and monitoring progress through PDSA cycle reviews and run charts
	Support	• Mentorship, support and ongoing assistance to monitor progress and review changes via iterative PDSA cycles and run charts • Considering to adopt the implemented changes (Change Package)
Maintenance	Support	• Mentorship, support and ongoing assistance are provided to monitor progress and review changes via iterative PDSA cycles and run charts • Considering to adopt the implemented changes (Change Package)
	Maintenance	• As for Support visits
	Action Learning Session	• Sharing change ideas and experiences with other facilities already randomised to the intervention

*CQI* Continuous Quality Improvement, *CRH* Centre for Rural Health, *PDSA* Plan-Do-Study-Act

CQI field activities for the entire project were scheduled in advance of the first intervention step. Clinic-based CQI activities occurred over 3 days of a given week, and administrative work scheduled for a separate day each week. CQI tools and principles (Tables 1 & 2) included Action Learning Sessions at the end of each transition phase to consolidate skills and share experiences. The intervention was based on the Institute for Healthcare Improvement (IHI) breakthrough collaborative CQI model [38]. Given time constraints imposed by the study design, activities mainly targeted the primary endpoint indicators: repeat HIV screening and HIV viral load (VL) testing according to South African PMTCT guidelines (Additional file 1: Text S1). Additional study endpoint indicators were addressed as time permitted.

**Table 2:** Description of CQI tools used in the intervention

CQI tool	Description
Process map	A map of clinic processes relating to a specific activity from the time a patient walks into the clinic A map, including waiting times and waiting site, is created for each activity as there may be differences in the process based on clinical need (e.g. a patient who is HIV-positive with tuberculosis co-infection versus a patient who is HIV-positive without tuberculosis). Mapping identifies areas for improved process efficiency, thereby reducing clinical workload and patient waiting times.
Run chart	A display of real-time data on target indicators The run chart is the essence of the ‘data-driven’ approach of CQI. Target indicators, or process indicators, are selected during the early stages of CQI implementation, for monitoring throughout the period of CQI activities. Facility staff can observe the results of changed activities using their own data in real-time on the so-called run chart. The run chart facilitates motivation and buy-in.
Fishbone diagram	A diagrammatic representation of factors contributing to a specific clinical endpoint – a ‘cause and effect’ diagram
PDSA cycle [44]	A representation of the process of change In the Planning stage, facility staff (guided by CRH CQI mentors) plan how to implement change targeting a specific endpoint (Plan). This is followed by actual implementation (Do) and review of progress and challenges (Study). The change becomes embedded in normal practice if implementation was successful, or generates a different method of implementation (Act).

*CQI* Continuous Quality Improvement, *CRH* Centre for Rural Health, *PDSA* Plan-Do-Study-Act

Prior to transition-phase CQI activities, a 2-week lead-in period (approximately 4 visits) was planned, to introduce the newly randomised cluster to the CRH team and CQI concepts. A standardized ‘dose’ of intensive CQI of ~ 19 visits, was planned for each transition step. A schedule of monthly follow-up (CQI maintenance) visits was also planned thereafter and varied by cluster due to order of randomisation.

Efforts to prevent cross-contamination whilst enabling buy-in for sustainability of CQI were made: only randomised clinics were invited to attend Action Learning Sessions, and PHC supervisors were excluded from these events during the intervention period (unlike ‘real-world’ implementation of CQI) as they supervise multiple clinics. However after the final transition step was complete, a final joint Action Learning Session with staff from all 7 clinics, PHC supervisors, sub-district and district DoH staff was held. In order to reduce bias the AHRI study investigators (evaluators) refrained from intervening in CQI clinic processes, although some operational co-involvement was required (e.g. introducing the CRH team to clinic staff).

#### Comparator

During the control exposure state, maternal and child healthcare providers continued providing DoH standard of care to ANC and PNC attendees as usually implemented. The South African maternal and child health strategic plan outlines the maternal package of services to be provided, including basic antenatal care. Usual training for staff involves weekly 1 hour in-service training on current evidence-based guidelines applied to primary healthcare services and may include training on ANC or PNC. The usual training, however, does not contain a mentoring component and is not a data driven process to evaluate the implementation of evidence-based guidelines. Additional background details on DoH standard of care are provided (Additional file 1: Text S1).

#### Data sources

All eligible women had their Maternity Case Record (MCR) photographed at delivery, excluding the intrapartum section, based on UKZN Biomedical Research Ethics Committee waiver of requirement for consent to access routine DoH data. All consenting women were interviewed at delivery and their infant’s Road-to-Health Booklet (RtHB) photographed. The structured interview covered themes on demographics, satisfaction with services, obstetric history, pregnancy intention, healthcare expenditures, access to care, and knowledge (infant feeding and HIV). At the 3–6 day and 6-week postnatal visits all consenting women were interviewed and their infant’s RtHB photographed. The structured interview at the 3–6 day visit was identical to the delivery visit. The 6-week structured interview covered themes on demographics, satisfaction with services, knowledge and uptake of services (HIV, PMTCT services, adherence to ART, contraception, self-reported infant feeding practices), healthcare expenditures, and access to care. All questionnaires contained questions in English with isiZulu translations and were conducted in isiZulu.

Structured interviews of consenting healthcare providers at the 7 study clinics were undertaken in English covering themes on job satisfaction, motivation and antenatal care practices, at study mid-point and study end.

Process evaluation data sources included semi-structured healthcare provider interviews undertaken in English, and detailed CQI implementation records from CRH for each PHC. The latter included actual visit dates, visit type, and descriptions of the successes and challenges the CRH CQI mentors encountered in implementing CQI.

Data collection from clusters commenced on 15 July 2015 and concluded on 30 January 2017. Each cluster contributed pre-exposure data until rolled over to the CQI intervention. Data collection continued from all clusters throughout the study until project end, providing pre-exposure, transition phase and post-exposure outcome data on all clusters.

As women were recruited at delivery or thereafter starting on 15 July 2015 - with retrospective capturing of their routine antenatal care data – the 2-month baseline data collection period contributed an additional observation period of ~ 6 months, resulting in a total data collection period of ~ 19 months and total observation period of ~ 25 months. The final post-exposure period (after all clusters had received the intervention) was 4.5 months (Fig. 2).

Eleven data clerks trained in International Conference on Harmonisation (ICH) Good Clinical Practice (GCP) guidelines were based at either Hlabisa Hospital or the 7 PHCs throughout the study. All data collected in clinics and Hlabisa Hospital, including cameras, were returned daily to the AHRI data centre for secure storage and capturing. A data capturing team of five trained in ICH GCP, including research nurses and quality controllers, captured clinical and laboratory data from digital photographs of MCRs and infant RtHB, and all information from structured questionnaires onto a REDCap™ study database [39].

#### Randomisation and blinding

Each clinic was a cluster, with the exception of the two smallest clinics, which jointly formed one cluster. After a baseline data collection period of 2 months, the first cluster crossed over from control to intervention exposure state on 29 September 2015; subsequently, one additional cluster crossed over every 2 months (Fig. 2). The six clusters were divided into 3 groups by patient volume (low, medium and high). To increase the likelihood that the sample sizes in intervention and control exposure states were similar, and to improve balance, we then randomised the clusters to the six calendar sequences of crossing over, such that both the first three and the last three sequences included one small, one medium, and one large cluster. A senior biostatistician performed randomisation for all clusters prior to the first intervention step. All study implementers, evaluators and clinic health workers were blinded to the initial randomisation status. Two weeks prior to each scheduled intervention step crossover date, the custodian of the randomisation list (AHRI Chief Information Officer) revealed the randomised cluster to the AHRI study team. The AHRI study team then introduced the CRH team to the randomised cluster for CQI training to commence.

### Analysis

#### Power calculation

For our baseline power calculation, we assumed – based on local routine primary care data – that without the MONARCH intervention viral load testing would be carried out in 40% of all pregnant women who were HIV-positive and repeat HIV testing would be carried out in 65% of all pregnant women who were HIV-negative. Based on local routine primary care data, we further assumed that half of all pregnant women would be HIV-positive and that pregnant women would make three ANC visits. We assumed an intracluster correlation coefficient (ICC) of 0.10. This coefficient is conservative compared to ICCs that we empirically measured in a similar setting (PHCs in sub-Saharan Africa providing ANC and HIV treatment and care), which ranged from 0.00 to 0.07 [40]. Finally, we assumed that we could not use information from 15% of enrolled women because of missing data. Given these assumptions, we estimated that we would have 80% power to detect at least a 15 percentage point increase in our two primary endpoints at the 5% significance level if we enrolled a total of 1260 pregnant women (i.e., 630 women who were HIV-positive and 630 women who were HIV-negative). Additional file 1: Table S1 shows the minimum detectable differences for this sample size with a number of alternative ICCs and endpoint values in the control exposure state.

#### Statistical analysis

The data will be analyzed by intention-to-treat (ITT). For ITT analyses, patient outcomes will be analyzed by the exposure status of the clinic attended at the first antenatal booking visit, independent of later clinic switches. We will analyse the CQI effect using mixed effects generalized linear regression models. In the main analysis, we will include a fixed effect for the time step and a random effect for clinic, following Hussey and Hughes [41], as well as adjust for clustering of standard errors at the clinic level. For the analyses of our two primary endpoints, which are binary, we will use modified Poisson regression [42] within the generalized linear regression framework. Our main results will thus be expressed as risk ratios. In sensitivity analyses, we will adjust for patient pre-exposure characteristics, and we will assess effect modification by secular time and time since intervention start in a clinic [43]. Stata (version 15.0, StataCorp, College Station, Texas) will be used for all analyses.

#### Process evaluation

For the *Process Evaluation* we will use a logic framework to explore the relationship between (i) input factors or resources that guide; (ii) activities needed to transform inputs into outputs processes; (iii) output elements comprising health service products produced with the inputs and activities; and (iv) the outcomes of this change process. A SPIRIT checklist pertaining to this protocol is attached (Additional file 2).

## Discussion

The MONARCH stepped-wedge RCT is the first trial to determine the effectiveness of an intervention to increase testing for health indicators among pregnant women that are critical for good health outcomes in HIV hyperendemic communities: VL testing among pregnant women who are HIV-positive and repeat HIV testing among pregnant women who are HIV-negative. The MONARCH study is also one of the first to measure the causal effect of CQI on ANC and PNC services in sub-Saharan Africa.

The MONARCH trial was embedded within the public health system and implemented within routinely available resources including the physical infrastructure of primary care clinics, data systems, and human resources. The success of the CQI intervention depends not only on this context, including staff motivation and the other resources available to change clinical processes. One of the advantages of CQI is that it leads to a selection of actions that can lead to successful improvements given the local abilities and constraints. Through local data collection and clinic-specific analysis health workers are empowered to identify the root causes of existing shortcomings; through small-scale tests in PDSA cycles they can develop the locally best solutions to address these shortcomings. The iterative manner in which CQI unfolds ensures that candidate solutions are repeatedly tested and modified until robust and sustainable approaches have been identified [44, 45]. Given significant resource constraints, it is particularly important to understand the mechanisms of the locally developed approaches and the determinants of their success. The Process Evaluation, which was nested in this trial, will therefore be highly informative in understanding the implementation of this complex intervention.

The MONARCH trial has several limitations. First, it took place in a real-life setting where normal service delivery and targets had to be met during CQI implementation – this may have limited the availability of healthcare providers to participate in the intervention. This test of the effectiveness of CQI was thus a test of CQI “in real life” and we cannot conclude from a null finding that CQI cannot be highly effective in a context with fewer outside pressures and more resource commitments. Second, advertisement of the study to DoH sub-district and facility staff prior to MONARCH study start may have driven changes towards endpoint targets and biased impact evaluation towards the null. Finally, the health workers in the control clusters might have learned about CQI methods and approaches to improve endpoint attainment from the health workers in the intervention clusters, also resulting in bias towards the null. Conversely, our efforts to reduce such contamination – such as blinding health workers to the randomisation status of their clinic and instructing the CQI mentors not to suggest solutions developed by previous clinic teams in their interactions with new clinic teams until post-randomisation – run counter to the philosophy of CQI, which includes sharing of solutions and approaches between CQI teams. These measures, which increase the validity of the trial, may have reduced the effectiveness of the CQI intervention that we have tested.

Overall, we expect that the results of the MONARCH trial and nested process evaluation will be useful for policy makers and practitioners seeking to increase the quality of care for pregnant women in HIV hyperendemic communities. It will also be useful for health systems managers striving to improve quality of primary care in severely resource-constrained clinics. Study results will be shared with local and regional policy makers during policy engagement workshops and presentations. We will also disseminate our results via peer-reviewed journals and presentations at scientific conferences.

### Trial status

The study commenced in mid-July 2015 and ended on 30 January 2017. At the time of submission of the manuscript, no other articles pertaining to the protocol are published or under consideration for publication.

## Additional files

Additional file 1: MONARCH SWT protocol. Text S1: South African National Department of Health standard of care: PMTCT guidelines and basic antenatal care. **Table S1.** Minimum detectable differences in percentage points by outcome. (DOCX 25 kb)

Additional file 2: MONARCH SWT protocol. Description of data: SPIRIT checklist. (DOCX 28 kb)

## Abbreviations

AHRI: Africa Health Research Institute
ANC: Antenatal care
ART: Antiretroviral therapy
CQI: Continuous Quality Improvement
CRH: Centre for Rural Health
DoH: Department of Health
GCP: Good Clinical Practice
HIV: Human Immunodeficiency Virus
ICC: Intra-cluster correlation coefficient
ICH GCP: International conference on harmonisation Good Clinical Practice (guidelines)
ITT: Intent-to-treat (analysis)
MCR: Maternity case record
MONARCH: Management and optimization of nutrition, antenatal, reproductive, child health and HIV care
PDSA: Plan-Do-Study-Act
PHC: Primary healthcare clinic
PIPSA: (AHRI) Population Intervention Platform Surveillance Area
PMTCT: Prevention of mother-to-child transmission of HIV
PNC: Postnatal care
QI: Quality Improvement
RtHB: Infant road-to-health booklet
VL: HIV viral load
