# Supplementary material for: The MONARCH intervention to enhance the quality of antenatal and postnatal primary health services in rural South Africa: protocol for a stepped-wedge cluster-randomised controlled trial
Source: BMC Health Serv Res. 2018 Aug 8;18:625. doi: 10.1186/s12913-018-3404-3 (PMC6083494; doi:10.1186/s12913-018-3404-3)
Supplement: Supplementary file 1 — MONARCH SWT protocol. Text S1: South African National Department of Health standard of care: PMTCT guidelines and basic antenatal care. Table S1. Minimum detectable differences in percentage points by outcome. (DOCX 25 kb) [file 12913_2018_3404_MOESM1_ESM.docx]

# Additional File 1

In this appendix, we provide additional details related to our study, including:

- Text S1: South African National Department of Health standard of care: PMTCT guidelines and basic antenatal care
- Table S1. Minimum detectable differences in percentage points by outcome

## Text S1. South African National Department of Health standard of care: PMTCT guidelines and basic antenatal care

As part of achieving health care equity, strengthening HIV prevention and treatment services is a priority area in South Africa which is the highest HIV burden country in the world.

*The basic antenatal care* approach recommends every woman attends antenatal care at least four times during her pregnancy, beginning in the first trimester to receive preventive services for maternal health, identify high risk pregnancies, and prepare for delivery and the postpartum period, including infant feeding advice. As per standard South African antenatal guidelines, all pregnant women are asked to return every six weeks until delivery or more frequently if the pregnancy is complicated [32].

For *pregnant/ breastfeeding women with unknown or negative HIV status*, South African PMTCT guidelines recommend provider-initiated counselling and testing (PICT) for HIV every 3 months during pregnancy, during labour/delivery, at the 6-week infant immunization visit and every 3 months during the breastfeeding period. Objectives are early diagnosis of HIV to shorten foetal/ infant exposure to maternal HIV viraemia, and improve maternal health. PICT is performed with a point-of-care rapid HIV test. After confirmation with a second rapid HIV test, immediate ART initiation (usually a fixed-dose combination, FDC, with tenofovir disoproxil fumarate, emtricitabine and efavirenz unless contraindicated) is recommended. Blood samples are drawn for CD4+ T-cell counts, haemoglobin, hepatitis B screening and serum creatinine. If the 2^nd^ confirmatory test is inconclusive a blood draw for HIV ELISA is recommended [33].

*Viral load monitoring of HIV-positive women* varies by ART status and VL result on ART. For *pregnant women initiating ART* for the first time (first-line regimen), a VL test is recommended 3 months after initiation, 6 months post-initiation then 6-monthly thereafter throughout pregnancy & breastfeeding if the VL is below 1000 copies/mL. The laboratory assay limit of detection of HIV is 40 copies/mL. For *pregnant women already on ART at first antenatal booking*, an immediate VL test is recommended regardless of last test date. If the VL is below 1000 copies/mL, repeat testing is recommended every 6 months thereafter throughout pregnancy and breastfeeding. If the VL is >1000 copies/mL, a repeat VL test is recommended 1 month later with adherence counselling. Subsequent VL testing is based on VL results. Persistent viraemia >1000 copies/mL requires an urgent switch to second-line therapy [33].

*HIV-exposed infants (HEI)* are required to be tested with HIV DNA PCR (dried blood spot) at birth and commence a minimum of 6 weeks’ prophylaxis with nevirapine (NVP) until confirmed HIV-negative. A repeat HIV test at 10 weeks (or 18 weeks for 12-week NVP prophylaxis) with HIV DNA PCR and a rapid HIV antibody test at 18 months, are recommended as a minimum. Additional testing is recommended for clinical suspicion of HIV infection, 6 weeks after cessation of breastfeeding, or death of an immediate family member attributable to HIV. Whether an HIV DNA PCR test or antibody test is performed depends on infant/child age: PCR if under 18 months, antibody of 18 months or older. HIV-positive infants must be commenced on ART immediately whilst awaiting results of confirmatory tests [33].

*The Maternity Case Record (MCR)* is the standardized official national document provided to all pregnant women in South Africa, regardless of public or private health care services [32]. Up to date documentation of the MCR is required as its function is to communicate between different facilities or levels of care received during pregnancy, and is retained by the mother until delivery – at delivery, the MCR is retained at the confinement facility [32].

## Table S1. Sample size calculation: minimal detectable differences for a sample size of 1260 women

|  | **ICC** | | | | |
| --- | --- | --- | --- | --- | --- |
| **Endpoint value in the control arm** | 0.01 | 0.05 | 0.10 | 0.15 | 0.20 |
| ***Viral load testing*** | | | | | |
| **30%** | 12.6 | 13.5 | 13.4 | 13.1 | 12.7 |
| **40%** | 13.5 | 14.4 | 14.3 | 14.0 | 13.6 |
| **50%** | 13.8 | 14.7 | 14.6 | 14.3 | 14.0 |
| ***Repeat HIV testing*** | | | | | |
| **50%** | 13.8 | 14.7 | 14.6 | 14.3 | 14.0 |
| **65%** | 13.2 | 14.0 | 13.9 | 13.6 | 13.3 |
| **80%** | 11.0 | 11.8 | 11.7 | 11.4 | 11.1 |

We show the minimal detectable differences for the two primary endpoints of the MONARCH stepped-wedge cluster-randomised controlled trial for a range of intracluster correlation coefficients (ICC) and endpoint values in the control exposure state. The minimal detectable difference is measured in percentage points.
